# *Sarocladium implicatum*: an unusual agent of opportunistic infection in a COVID-19 patient

**DOI:** 10.1590/S1678-9946202567075

**Published:** 2025-11-03

**Authors:** Gilberto Gambero Gaspar, Ludmilla Tonani, Patrícia Helena Grizante Barião, Otávio Guilherme Gonçalves de Almeida, Roberto Martinez, Marcia Regina von Zeska Kress

**Affiliations:** 1 Universidade de São Paulo, Faculdade de Medicina de Ribeirão Preto, Departamento de Medicina Social, Ribeirão Preto, São Paulo, Brazil; 2 Universidade de São Paulo, Faculdade de Ciências Farmacêuticas de Ribeirão Preto, Departamento de Análises Clínicas, Toxicológicas e Bromatológicas, Ribeirão Preto, São Paulo, Brazil; 3 Universidade de São Paulo, Faculdade de Medicina de Ribeirão Preto, Departamento de Clínica Médica, Ribeirão Preto, São Paulo, Brazil

**Keywords:** Sarocladium implicatum, Immunocompromised, COVID-19, Hyalohyphomycosis

## Abstract

*Sarocladium* spp. are filamentous fungi commonly associated with plant diseases and only rarely cause hyalohyphomycosis in humans. Immunosuppressed patients are at risk for this infection, which typically presents with skin and subcutaneous lesions that may eventually disseminate to internal organs. This study reports a case of a man in intensive care following SARS-CoV-2 infection. During hospitalization, he developed neutropenia, persistent fever, and a cavitary lung lesion. *Sarocladium* spp. was isolated from blood cultures, and the patient was treated with voriconazole, leading to a successful cure. To our knowledge, this is the first reported case of *Sarocladium implicatum* infection in a COVID-19 patient, underscoring the importance of monitoring opportunistic fungal infections in immunocompromised individuals, particularly during epidemics and pandemics.

## INTRODUCTION

The genus *Sarocladium* includes fungal species widely distributed in the environment and traditionally associated with plant infections. *Sarocladium* spp. belongs to the order *Hypocreales* and are taxonomically classified within the highly diverse genus *Acremonium* ^1^. Due to taxonomic similarities and reclassifications, several important human pathogenic species have been renamed, such as *Sarocladium strictum* (formerly *Acremonium strictum*) and *Sarocladium kiliense* (formerly *Acremonium kiliense*)^2^.

Infections caused by *Sarocladium* and *Acremonium* are diverse, ranging from localized conditions (e.g., onychomycoses and keratitis) to invasive infections, such as fungemia and disseminated infections, particularly in immunocompromised patients^3^. The identification of these species is challenging, often delaying rapid diagnosis and complicating patient management. Moreover, molecular methods are essential to differentiate *Sarocladium* from other opportunistic fungi, such as *Fusarium* spp^3^.

Infection with SARS-CoV-2 can result in severe complications, leading to critical illness and the need for intensive care. In these patients, opportunistic fungal infections may emerge due to pathophysiological changes associated with COVID-19, including an exacerbated inflammatory response, adverse effects of therapeutic interventions, and disruption of the integumentary barrier caused by medical devices such as mechanical ventilation and catheters. Opportunistic fungi are a common cause of pulmonary infections in COVID-19 patients, with notable pathogens including *Aspergillus* spp., Mucorales, *Cryptococcus* spp.**,** *Histoplasma capsulatum*, *Pneumocystis jirovecii*, *Fusarium* spp., among others^4^.

To date, no *Sarocladium implicatum* infection cases have been reported in patients with SARS-CoV-2 infection. This study describes the first documented case of lung and bloodstream infection caused by *Sarocladium* spp. in a patient with COVID-19.

### Ethics

The study was conducted with the approval of the Research Ethics Committee of both Hospital das Clinicas da Faculdade de Medicina de Ribeirao Preto – Universidade de Sao Paulo (Nº 7.357.949) and Faculdade de Ciencias Farmaceuticas de Ribeirao Preto – Universidade de Sao Paulo (Nº 561/2021).

## CASE REPORT

A 64-year-old Brazilian man, residing in the Northeastern region of Sao Paulo State, Brazil, presented with a history of chronic smoking and presumed chronic obstructive pulmonary disorder (COPD). At age 53, he had a penile lesion, which was excised at another medical facility and histologically diagnosed as Kaposi’s sarcoma.

**Figure 1:**
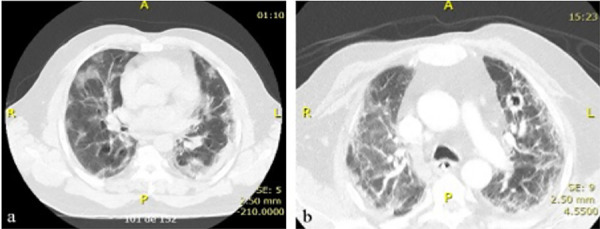
Computed tomography of the patient’s chest: (A) At hospital admission due to COVID-19; (B) At the time of isolation of *Sarocladium* spp. from the patient’s bloodstream.

In May 2021, he was admitted to the hospital with a four-day history of fever, malaise, anoxemia, and progressive dyspnea, particularly during physical activity (Figure 1). His oxygen saturation on room air was 84%. Oxygen therapy was initiated at 3 liters per minute via nasal cannula, which improved his oxygen saturation to 90%. On physical examination, he was in good general condition, weighting 106 kg, afebrile, with a respiratory rate of 18 breaths per minute. Chest auscultation revealed diffuse bilateral pulmonary rales.

A computed tomography (CT) scan of the chest showed diffuse interstitial opacities and areas of alveolarization with bilateral ground-glass attenuation, affecting approximately 40%–50% of the lung parenchyma (Figure 1A). A real-time polymerase chain reaction (RT-PCR) test on a respiratory secretion sample was positive for SARS-CoV-2. An ELISA test for anti-HIV antibodies was negative. The diagnosis of COVID-19 and possible bacterial pneumonia led to treatment with ceftriaxone, azithromycin, and methylprednisolone (2 mg/kg/day).

Despite treatment, the patient’s clinical condition deteriorated within hours, requiring transfer to the intensive care unit (ICU). Orotracheal intubation and noradrenaline administration for blood pressure support were required. Laboratory results revealed elevated creatine phosphokinase (CPK, 1,800 U/l), indicative of rhabdomyolysis, and acute kidney injury due to urinary catheter obstruction. Additionally, episodes of sinus tachycardia were managed with amiodarone, while hyperglycemia was controlled with daily insulin administration.

Over the course of hospitalization, the patient’s condition gradually improved. Antimicrobials were discontinued on day 7, methylprednisolone was tapered to 1 mg/kg/day, and the tracheal tube was removed on day 10, with oxygenation maintained via nasal cannula.

On day 14, the patient experienced clinical deterioration with fever and septic shock, attributed to nosocomial pneumonia. *Klebsiella pneumoniae* and *Enterococcus faecalis* were isolated from tracheal secretions, prompting treatment with meropenem and vancomycin. His respiratory status worsened, requiring re-intubation, mechanical ventilation, and prone-positioning sessions. Methylprednisolone at 125 mg/day was continued for eight days before gradual tapering.

Fever persisted over following days, but hemodynamic recovery and improved blood oxygenation enabled tracheal extubation on day 24. Mechanical ventilation was replaced with non-invasive ventilation. However, the patient developed severe neutropenia: during the first two weeks of hospitalization, leukocytes count fluctuated around 15,000 cells/µL but dropped to 1,700 cells/µL on day 26, with complete absence of neutrophils in peripheral blood. Neutropenia was attributed to vancomycin and meropenem, which were subsequently discontinued. Filgrastim was initiated, leading to gradual recovery of circulating neutrophils. Given the hypothesis of opportunistic cytomegalovirus (CMV) infection, the patient received ganciclovir after PCR analysis detected 5,287 viral copies/mL in the blood.

**Figure 2:**
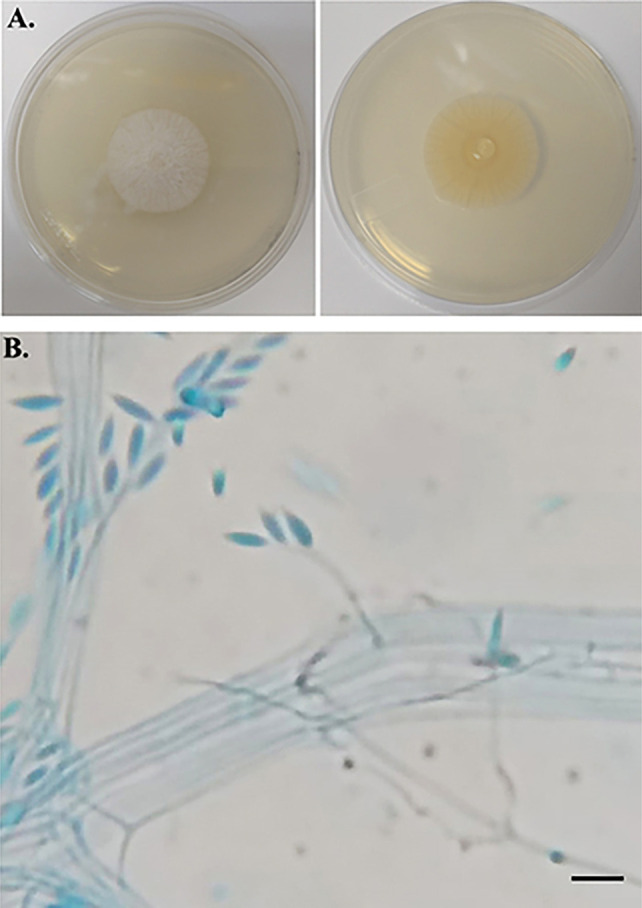
Macro and micromorphology of the isolated filamentous fungus: (A) Fungal colony on potato dextrose agar (PDA) media culture at 28 °C for 12 days. Left panel: verse of the colony (white, raised, and downy); right panel: reverse of the colony (pale orange); (B) Micromorphology of *Sarocladium implicatum* (lactophenol cotton blue preparation, ×1000) showing erect, simple, hyaline, smooth-walled, solitary conidiophores (15–30 μm long, 1–2 μm wide at the base). Distinct periclinal thickening of the conidiogenous locus. Conidia are unicellular, fusiform with sharply pointed end (5–8 × 1–2 μm), hyaline, and thin-walled. Scale bar 10 µm.

Fluconazole was prescribed for 10 days to treat urinary candidiasis following the detection of *Candida parapsilosis* (90,000 CFU/mL) in a urinary sample. Despite treatment, the patient continued to experience fever peaks ranging from 38.0 °C to 38.5 °C. Blood culture performed on day 29 of hospitalization suggested the presence of a filamentous fungus (Figure 2). Molecular analysis based on sequencing of the internal transcribed spacer (ITS) region of ribosomal DNA^5^ identified the fungus as *Sarocladium implicatum* (Table 1), showing 100% identity with the GenBank reference sequence HG965021.1 (E-value = 0.0). The corresponding ITS sequence has been deposited in GenBank under accession Nº PV698332. The isolate is preserved in the culture collection of the Laboratory of Gene Expression and Proteomics of Filamentous Fungi (FCFRP-USP) under code LMC21301.01. Additionally, susceptibility testing following the M38-A3 protocol (CLSI, 2017) was performed to determine the minimum inhibitory concentration (MIC) of antifungal agents, with results presented in Table 1.

**Table 1:** Molecular identification and Minimum Inhibitory Concentration (MIC) of antifungals (μg/mL)

Clinical isolate	ITS*				MIC (µg/mL)			
	Species	GenBank Ref. Seq.	Identity (%)	E-value	AMB	VOR	ITR	TBF
LMC 21301.01	*Sarocladium implicatum*	HG965021.1	100	0.0	4	8	16	>128

ITS = internal transcribed spacer of ribosomal DNA; *PCR amplification and sequencing with primers ITS1 and ITS4; MIC = minimum inhibitory concentration; AMB = amphotericin B; VOR = voriconazole; ITR = itraconazole; TBF = terbinafine; LMC = clinical mycology laboratory.

On day 31, signs of bacteremia—such as persistent fever, chills, hypotension, and elevated inflammatory markers (e.g., C-reactive protein)—prompted the collection of blood cultures and initiation of empirical antibiotic treatment. A new chest CT scan revealed ground-glass and reticulated opacities, as well as bands of atelectasis diffusely distributed throughout the lungs. A cavitary lesion measuring 1.7 cm in diameter was also observed in the upper left lobe (Figure 1B).

While the patient experienced clinical improvement, fever persisted along with dry cough and tachypnea (respiratory rate of 28 breaths/min and oxygen saturation of 89% with oxygen administration at 3 liters per minute via a nasal cannula). Voriconazole (200 mg orally every 12 hours) was initiated on day 40 of hospitalization and continued for 15 days. Subsequent blood cultures showed no growth of filamentous fungi, and the patient was discharged.

Follow-up chest CT scans performed six and 15 months after the isolation of *Sarocladium implicatum* demonstrated resolution of pulmonary cavitation and significant reduction of diffuse opacities observed in earlier images. At the one-year follow-up, the patient was well and eupneic, with a persistent dry cough and crackles at the lung bases.

## DISCUSSION

*Sarocladium* species are recognized as phytopathogens and occasional human pathogens, frequently associated with hyalohyphomycosis. Clinical manifestations often follow traumatic inoculation and may slowly evolve over months or even years. In other cases, infections are more aggressive, spreading to contiguous tissues or distant sites^6^. Within the genus, the main pathogenic species for humans are *S. kiliense* and *S. strictum*, with reports of central nervous system infections, cutaneous and subcutaneous infections, disseminated infections, endocarditis, fungemia, gastrointestinal infections, joint and bone infections, keratitis, mycetoma, onychomycoses, peritonitis, and respiratory infections. Severe infections typically occur in immunocompromised patients^3^.

*Sarocladium* (formerly *Acremonium*) *implicatum* has been associated with various infections and isolated from clinical samples, including sputum, bronchial washing, sinuses, bronchoalveolar lavage (BAL), bone, and skin^7-9^. This species exhibits lower virulence in immunosuppressed murine model compared to *S. kiliense* and *S. strictum* ^10^, which may explain the lower number of reported cases attributed to *S. implicatum*.

In this case, the patient initially presented pulmonary complications due to SARS-CoV-2 infection, followed by immunosuppression resulting from corticosteroid therapy, hyperglycemia, neutropenia, and vancomycin use. Bloodstream infections caused by *Sarocladium* spp. in neutropenic patients undergoing invasive procedures have been previously reported^11-13^. Lung infections attributed to *Sarocladium* spp. or *Acremonium* spp. have also been associated with patient comorbidities, such as lung cancer, chronic granulomatous disease, and solid organ transplants (e.g., kidney, lung, or liver). These patients often exhibited prolonged respiratory symptoms, with radiological findings including consolidations, nodules, and/or cavitated nodules^14,15^. In one report, a fungal isolate was recovered from a necrotic neoplastic mass obstructing the bronchus^7^. Cavitated nodules observed in lung imaging of critically ill COVID-19 patients should raise suspicion of opportunistic fungal infections, among other diagnostic hypotheses^16^.

In this case, the persistence of fever despite the broad-spectrum antibacterial, antifungal (fluconazole), and antiviral (ganciclovir) therapy prompted periodic blood sampling for microbiological surveillance. Isolation of a filamentous fungus from blood cultures, along with the identification of a cavitated pulmonary nodule in a neutropenic patient, initially suggested aspergillosis or opportunistic fusariosis. However, both morphological and molecular analyses confirmed the presence of *S. implicatum*.

Fluconazole therapy was initiated on day 30 and continued until day 40 of hospitalization, beginning one day after the collection of the blood culture that yielded *S. implicatum*. Consequently, this case does not meet the criteria for a breakthrough fungal infection.

Antifungal susceptibility testing showed a MIC of 4 µg/mL for amphotericin B and 8 µg/mL for voriconazole. Although the voriconazole MIC suggests reduced susceptibility, this antifungal was selected based on clinical availability. Notably, the patient showed marked clinical improvement in conjunction with neutrophil recovery, suggesting that immune reconstitution was critical in treatment response despite the elevated MIC. The favorable clinical outcome observed aligns with previous reports of successful treatment of systemic hyalohyphomycosis caused by *Acremonium* spp. or *Sarocladium* spp., managed with amphotericin B, various azoles, and other antifungal agents^6^.

Although the patient had a history of Kaposi’s sarcoma, it occurred more than a decade ago with no subsequent recurrence. During the current hospitalization, further immunological investigations were limited by the acute clinical context. Nevertheless, HIV ELISA was negative, and the patient exhibited a rapid hematological recovery, arguing against chronic or AIDS-related immunosuppression. No additional causes of immunosuppression were suspected beyond those related to COVID-19 and its treatment, particularly severe neutropenia, corticosteroid therapy, and transient hyperglycemia. At a follow-up in 2024, the patient presented normal hematologic parameters and complete healing of the mucosa previously affected by Kaposi’s sarcoma, with no clinical or laboratory evidence of ongoing immunosuppression.

Due to the patient’s unstable condition and multiple co-infections, repeated blood cultures and invasive procedures such as bronchoscopy or biopsy were not performed. Nevertheless, the isolation of a filamentous fungus from the bloodstream in a neutropenic patient, along with compatible clinical and radiological findings and molecular identification as *S. implicatum*, strongly supports its etiological role. Pulmonary aspergillosis was initially considered; however, *Aspergillus* spp. was not isolated from any of the five tracheobronchial aspirate cultures collected over a 19-day period. Anti-*Aspergillus* antibody testing was negative both 17 days after fungal isolation and at the seven-month follow-up. While other causes of pulmonary cavitation cannot be entirely ruled out, the temporal correlation between *Sarocladium* isolation, clinical worsening unresponsive to antibacterial therapy, and the development of a cavitary lesion suggests that *S. implicatum* was the most likely opportunistic pathogen in this case.

COVID-19 infection creates a permissive environment for opportunistic fungal infections. Several mechanisms, including hyperinflammation induced by the host’s immune response, increase cell permeability and facilitate fungal invasion^17^. Environmental fungi, including hyalohyphomycosis-associated species, have been implicated in nosocomial infections in immunocompetent COVID-19 patients in ICUs. These cases are often associated with corticosteroid therapy or comorbidities such as morbid obesity^18^. A case of *S. kiliense* infection was described in an older diabetic woman receiving corticosteroids and antibiotics, who unfortunately did not survive despite treatment with liposomal amphotericin B^19^.

## CONCLUSION

The case reported in this study is apparently unprecedented, as no prior reports have described an opportunistic infection caused by *S. implicatum* in a COVID-19 patient. Bloodstream and lung infections due to *S. implicatum* are uncommon among human infections by *Sarocladium* spp. Given the impact of COVID-19, host immunosuppression, and ICU-related factors such as mechanical ventilation^17^, the emergence of *Sarocladium* species as nosocomial pathogens is plausible. This is the first identification of *S. implicatum* as a nosocomial infectious agent in a COVID-19 patient. Routine diagnostic analyses may misidentify *Sarocladium* spp. due to their morphological similarity to *Acremonium* spp. and *Fusarium* spp^3^. Thus, molecular identification, together with traditional culture-based techniques, is essential for accurate pathogen identification.

Although infections by *S. implicatum* are rare, hospital laboratories must be equipped to accurately identify these fungi to facilitate prompt and appropriate diagnosis and treatment for clinicians managing these often-overlooked opportunistic pathogens.

## Data Availability

The complete anonymized dataset supporting the findings of this study is included within the article itself.
